# Modifiable Risk Factors and Cognitive Function in Older Adults: A study from an Aging Cohort in Thailand

**DOI:** 10.1002/alz70860_102919

**Published:** 2025-12-23

**Authors:** Rapas Samalapa, Aisawan Petchlorlian

**Affiliations:** ^1^ Geriatric Excellence Center, King Chulalongkorn Memorial Hospital, The Thai Red Cross Society, Bangkok, Thailand; ^2^ Faculty of Medicine, Chulalongkorn University, Bangkok, Thailand

## Abstract

**Background:**

The increasing prevalence of dementia is a significant public health concern. Cognitive function is influenced by various risk factors. Identifying the modifiable risk factors within our population could lead to effective strategies to prevent dementia.

**Method:**

We conducted a study on an aging cohort at King Chulalongkorn Memorial Hospital, focusing on older adults aged 60 and older without dementia. This study included patients with available MoCA scores and data on relevant risk factors from October 2018 to July 2024. Cognitive outcomes were assessed using MoCA scores as continuous variables and categories (MoCA <25 for cognitive impairment, decline ≥2 points for cognitive decline). Participants with available follow‐up MoCA scores were included in the analysis of factors associated with cognitive decline. A random forest model was used to evaluate the relationships between risk factors and cognitive outcomes.

**Result:**

A total of 8,949 participants were included, with a mean age of 67.69 ± 21.33 years, and 75.75% were female. The mean baseline MoCA score was 25.99 ± 2.81, and 24.73% of participants had MoCA <25. The median follow‐up time was 3.2 years (IQR: 2.24–4.08). Of these, 2,655 participants with follow‐up MoCA scores were included in the analysis of cognitive decline. As continuous data, risk factors related to baseline MoCA scores, ranked by percentage increase in mean squared error (%IncMSE), included less education, obesity (waist‐to‐height ratio), hypertension, and social isolation. Factors associated with MoCA decline during follow‐up included hypertension, low physical activity and obesity. As categorical outcomes, less education, hypertension, obesity, and social isolation were associated with baseline MoCA <25. Factors related to a decline in MoCA score ≥2 during follow‐up included social isolation, hypertension, and low physical activity.

**Conclusion:**

This study identifies key modifiable risk factors influencing cognitive function in older adults in Thailand. Hypertension, obesity, and social isolation were associated with both baseline MoCA scores and cognitive decline over time. Additionally, lower educational attainment was linked to lower baseline MoCA scores, while low physical activity was specifically associated with cognitive decline. These findings support targeted interventions to address these risk factors and promote cognitive health in aging populations.

